# Infection to hypertension: a review of post-COVID-19 new-onset hypertension prevalence and potential underlying mechanisms

**DOI:** 10.3389/fcvm.2025.1609768

**Published:** 2025-08-18

**Authors:** Azin Teymourzadeh, Dmitry Abramov, Sayna Norouzi, Dennis Grewal, Giv Heidari-Bateni

**Affiliations:** ^1^ Department of Cardiovascular Medicine, Mayo Clinic, Rochester, MN, United States; ^2^ Division of Cardiology, Department of Medicine, Loma Linda University Medical Center, Loma Linda, CA, United States; ^3^ Division of Nephrology, Department of Medicine, Loma Linda University Medical Center, Loma Linda, CA, United States; ^4^ Division of Cardiology, Arrowhead Regional Medical Center, California University of Science and Medicine, Colton, CA, United States

**Keywords:** post-COVID complications, new-onset hypertension, angiotensin-converting enzyme pathway, inflammation, endothelial dysfunction

## Abstract

Post-COVID new-onset hypertension (PCNH) is an increasingly reported complication among COVID-19 survivors. PCNH can emerge up to 12 months postinfection, with elevated risks observed among older patients, particularly those who experienced severe COVID-19, and among females, implicating the possibility of age and hormonal influence. Leading theories converge on enduring dysregulation of the angiotensin pathway and endothelial dysfunction. In addition to renin–angiotensin alterations, sustained inflammation, lung vascular damage, deconditioning, and mental health decline may also impact the likelihood of PCNH. Conventional renin–angiotensin system (RAS) antagonists may help improve pathway distortions, while novel anti-inflammatory agents and recombinant ACE2 biologics can help mitigate endothelial injury to alleviate cardiovascular burden. This review highlights the multifaceted mechanisms driving PCNH and the need to elucidate timing, predictors, pathophysiology, and tailored interventions to address this parallel pandemic among COVID-19 survivors.

## 1 Introduction

The global COVID-19 pandemic has had a profound impact on public health, causing widespread physical and psychological consequences. With over 6.5 million deaths worldwide and immediate effects on different systems of the human body, the pandemic has raised major concerns globally (1). More recently, as a result of worldwide vaccination and a substantial decrease in the mortality rate, researchers are working to improve the understanding of the potential long-term health effects on survivors.

One area of growing concern is the development of post-COVID-19 new-onset hypertension (PCNH) in individuals who have recovered from COVID-19. Initial evidence suggests that even after complete remission from COVID-19 disease, some patients may be diagnosed with hypertension for the first time (2, 3) or experience a worsening of preexisting hypertension (4). Studies reported a significant prevalence of PCNH in patients who recovered from COVID-19. Additionally, we observed an increase in cardiovascular mortality associated with hypertensive disease in the first year of the pandemic (5, 6).

Understanding the mechanisms underlying COVID-19-induced hypertension is crucial for effective management and treatment strategies. In this review, we aim to explore the current literature surrounding the development of hypertension following COVID-19 infection, including potential mechanisms, risk factors, clinical implications, and therapeutic interventions. By understanding this emerging issue, we can better support the long-term health of COVID-19 survivors, particularly from a hypertension standpoint.

## 2 Method and search strategy

We conducted a comprehensive literature search using PubMed/MEDLINE and Embase databases from December 2019 to December 2024 to capture all relevant publications since the emergence of COVID-19.

### 2.1 Search strategy and terms

The search was performed using various combinations of the following Medical Subject Headings (MeSH) terms and keywords: “COVID-19,” “SARS-CoV-2,” “coronavirus,” “hypertension,” “blood pressure,” “new-onset hypertension,” “post-COVID,” “long COVID,” “post-acute COVID-19 syndrome,” and “COVID-19 sequelae.” Additional search terms included “post-COVID new-onset hypertension (PCNH),” “cardiovascular complications,” “renin-angiotensin system,” “ACE2,” “endothelial dysfunction,” and “systemic inflammation.” Boolean operators (AND, OR) and truncation symbols were used to capture variations in terminology and spelling.

### 2.2 Study selection criteria

We included peer-reviewed original research articles, systematic reviews, meta-analyses, and case series published in English that reported on hypertension development following COVID-19 infection. Studies were included if they (1) involved human subjects with confirmed COVID-19 diagnosis, (2) reported new-onset hypertension or blood pressure changes postinfection, (3) had follow-up periods of at least 30 days post-acute infection, and (4) provided quantitative data on prevalence, incidence, or risk factors. We excluded case reports with fewer than 10 patients, conference abstracts without full-text availability, preprints without peer review, and studies focusing solely on preexisting hypertension management during acute COVID-19.

### 2.3 Additional sources

We supplemented our database searches with manual screening of reference lists from included studies and relevant review articles. We also searched clinical trial registries (ClinicalTrials.gov, WHO International Clinical Trials Registry Platform) for ongoing studies. Gray literature was examined through Google Scholar and relevant professional organization websites to identify additional reports and guidelines.

## 3 Prevalence of post-COVID hypertension

The emergence of post-COVID-19 hypertension represents one of the most compelling cardiovascular sequelae of the pandemic. Multiple large-scale studies have demonstrated a consistent pattern of increased hypertension risk following COVID-19 infection, with evidence spanning millions of patients worldwide.

Meta-analyses encompassing over 19 million individuals reveal that COVID-19 survivors face a 65%–70% increased risk of developing new-onset hypertension compared with uninfected controls (7). The clinical magnitude of this phenomenon is substantial, with studies reporting PCNH rates ranging from 10.85% in non-hospitalized patients to 20.6% in hospitalized COVID-19 survivors within 6 months of recovery (8). Recent longitudinal population-based studies demonstrate a dramatic temporal increase in hypertension incidence during the pandemic years, with rates more than doubling from pre-pandemic levels and continuing to rise through 2023 (13).

The temporal dynamics of PCNH reveal additional complexity, with peak risk occurring within 30 days postinfection, though some studies suggest this risk may normalize by 91–120 days (9). Beyond new-onset cases, COVID-19 appears to exacerbate preexisting hypertension, with over half of previously hypertensive patients experiencing worsened blood pressure control post-recovery (4). Healthcare practitioners globally report increased diagnoses of new-onset hypertension in COVID-19 survivors, with some studies documenting PCNH in up to one-third of patients at 1-year follow-up (10, 11).

Collectively, these findings establish PCNH as a significant and persistent cardiovascular legacy of COVID-19, affecting millions of survivors worldwide and reshaping our understanding of post-viral cardiovascular disease. Detailed study characteristics and findings are presented in Table 1.

**Table 1:** Studies reporting post-COVID-19 new-onset hypertension.

Study	Population	Sample size	Follow-up	Key findings
Delilac et al. (4)	Hypertensive COVID patients	32	NR	Worsened hypertension control: 17/32 (53%) showed worsened BP control post-COVID
Zuin et al. (7)	Meta-analysis, five population studies	>19 million	7 months	New-onset hypertension: 12.7/1,000 COVID vs. 8.17/1,000 controls (HR: 1.7, 95% CI: 1.46–1.97, *p* < 0.0001)
Zhang et al. (8)	COVID-19 vs. influenza patients	NR	6 months	New-onset hypertension: 20.6% hospitalized, 10.85% non-hospitalized COVID patients; higher than influenza
Chevinski et al. (9)	COVID-19 survivors	NR	30–120 days	New-onset hypertension: OR 2.3 at 30 days, returned to baseline by 91–120 days
Vyas et al. (10)	COVID-19 survivors	248	1 year	New-onset hypertension: 32.3% (*n* = 80) developed
Krishnakumar et al. (11)	Healthcare practitioner survey	NR	NR	New-onset hypertension: 66% of practitioners reported increased cases in COVID survivors
Angeli et al. (12)	Pooled analysis, four studies	NR	NR	New-onset hypertension: 65% increased risk (OR: 1.65, 95% CI: 1.34–2.05); 9% COVID vs. 5% controls
Trimarco et al. (13)	Longitudinal cohort	>200,000	7 years	New-onset hypertension incidence: 2.11/100 person-years (2017–2019) → 5.20/100 (2020–2022) → 6.76/100 (2023)
Kazemi et al. (14)	Hospitalized COVID-19 patients, Iran	690	During hospitalization	New-onset hypertension: 67 patients (10%)
Azami et al. (15)	Non-hospitalized COVID-19 patients, Iran	5,355	12.5 ± 0.4 months	New-onset hypertension: 408 patients (17%) Total new/exacerbated hypertension: 864 patients (16%)
Daugherty et al. (16)	UnitedHealth Group, adults 18–65	193,113	4 months	New-onset hypertension: HR 1.56 (95% CI: 1.48–1.65), RD 1.10 (0.88–1.32) per 100 people vs. 2020 controls
Abdulan et al. (17)	Post-COVID patients, Romania	70	30 days	Worsened hypertension: Pre-COVID 68.57% → post-COVID 90% (*p* = 0.005) Risk factors: age, female gender, elevated BMI
Cohen et al. (18)	Medicare Advantage adults ≥65 years	87,337 COVID+ matched pairs	120 days post-acute	New-onset hypertension: RD 4.43 (2.27–6.37) per 100 patients vs. 2020 controls; HR 1.76 (1.58–1.97)
Mizrahi et al. (19)	Israeli healthcare members, mild COVID-19	299,870 matched pairs	30–360 days	New-onset hypertension: HR 1.16 (1.05–1.27), RD 8.3 (2.4–14.1) per 10,000 patients in late period (180–360 days)

BP, blood pressure; NR, not reported; RD, risk difference.

## 4 Contributing risk factors

The risk factors for the development of new-onset hypertension are not yet fully understood; however, meta-analysis has indicated a higher prevalence among women (*p* = 0.03) (7). This finding contrasts with the higher number of recorded COVID-19 cases and hospital admissions in men (20). Moreover, older age (*p* = 0.001) and history of cancer (*p* < 0.0001) were significantly associated with a higher risk of PCNH. Al-Aly et al. and Tisler et al. reported a significantly increased risk of hypertension in hospitalized and ICU-admitted patients compared with the non-hospitalized subjects. These results can indirectly indicate the role of the severity of the infection in the increased risk of PCNH (3, 21). On the other hand, there are conflicting data regarding the relationship between PCNH and some of the biomarkers of severity in severe acute respiratory syndrome coronavirus 2 (SARS-CoV-2) infection, including variations in cycle threshold (CT) scores and C-reactive protein (CRP) levels (10, 22).

## 5 Probable engaged pathways

**Figure 1:**
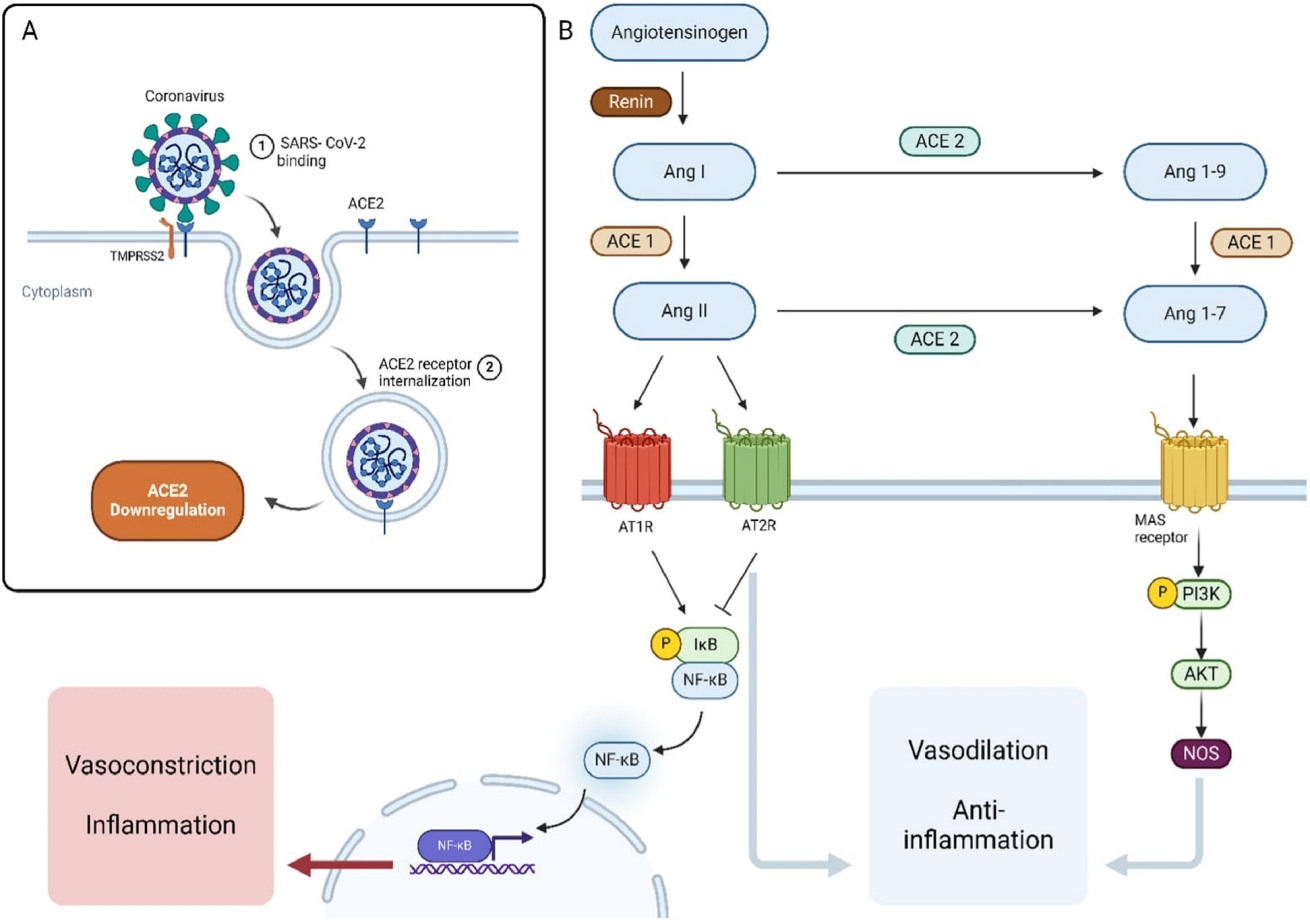
Mechanisms of SARS-CoV-2 Interaction with the Renin-Angiotensin System (RAS) and Its Downstream Effects. **(A)** SARS-CoV-2 binds to the ACE2 receptor on the host cell surface, leading to receptor internalization and downregulation of ACE2 expression. **(B)** The RAS pathway involves the conversion of angiotensinogen to Angiotensin I (Ang I) by renin, and then to Angiotensin II (Ang II) by ACE1. Ang II can bind to AT1R, promoting vasoconstriction and inflammation via the NF-κB pathway. Alternatively, Ang II can be converted by ACE2 into Ang 1–7, which acts through the MAS receptor to promote vasodilation and anti-inflammatory effects through the PI3K/AKT/NOS signaling pathway. ACE2 also converts Ang I to Ang 1-9, which can be further processed by ACE1.

SARS-CoV-2 enters the cell by binding to the angiotensin-converting enzyme 2 (ACE2) receptor using the spike (S) protein. The membrane fusion requires the S protein to bind to both ACE2 and TMPRSS2 (23). Co-expression of ACE2 and TMPRSS2 is particularly high in four types of cells: type II pneumocytes, ileal absorptive enterocytes, endothelial smooth muscle cells, and nasal goblet secretory cells (24). These cells serve as primary targets for the SARS-CoV-2 virus. Once the virus enters the cell, it exerts various effects in two major pathways discussed in more detail below (Figure 1).

### 5.1 ACE pathway

#### 5.1.1 ACE1 and ACE2 balance

**Figure 2:**
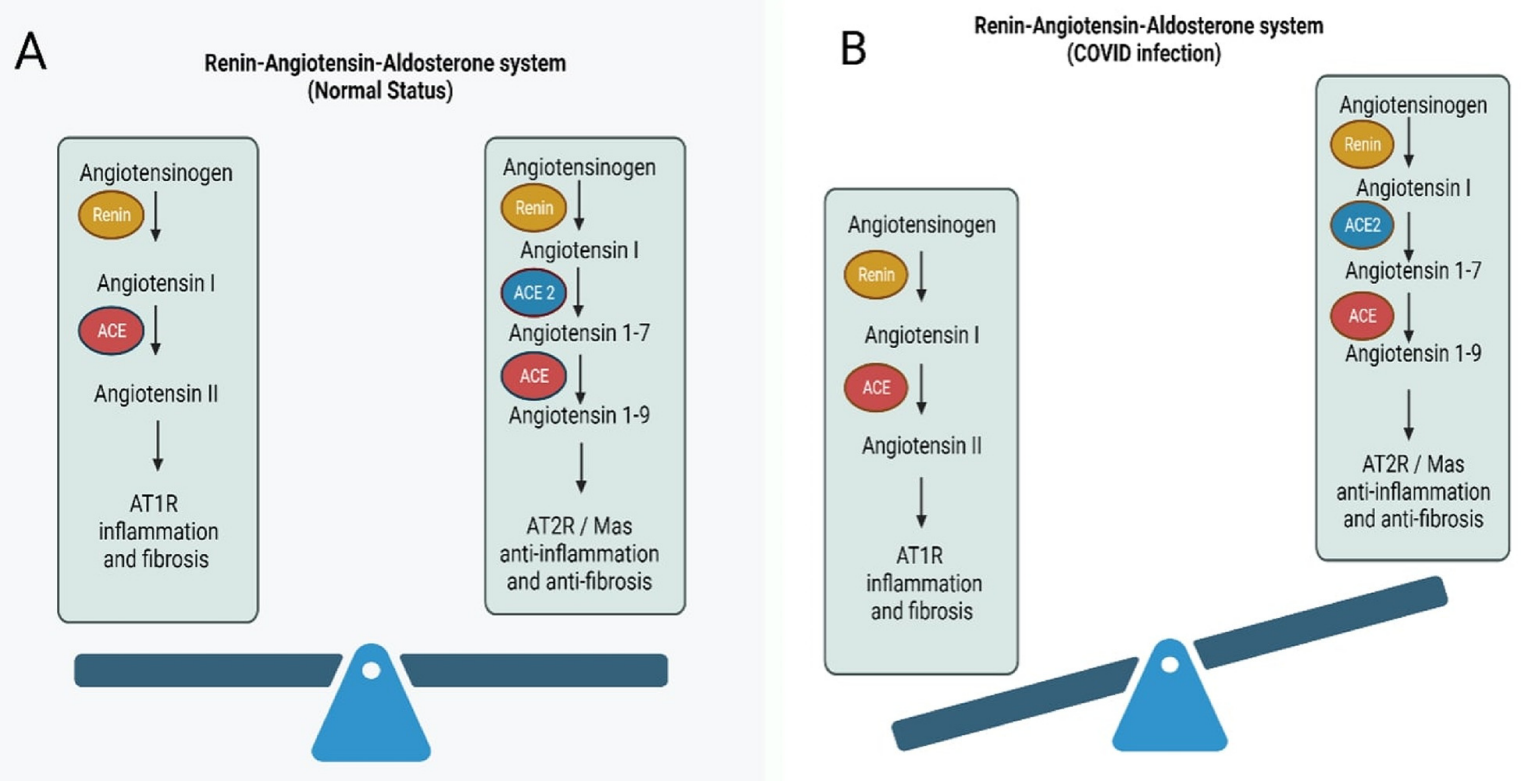
Disruption of the Renin-Angiotensin-Aldosterone System (RAAS) During COVID-19 Infection. **(A)** Under normal conditions, the RAAS maintains a balance between pro-inflammatory and anti-inflammatory signals. **(B)** During COVID-19 infection, ACE2 is downregulated following SARS-CoV-2 binding, reducing the conversion of Angiotensin I and II to their protective forms.

ACE1 converts angiotensin I to angiotensin II, driving vasoconstriction and increasing blood pressure (25, 26). Its overactivity also induces inflammation and fibrosis via increasing the reactive oxygen species (27, 28). In contrast, ACE2 inactivates angiotensin II into anti-inflammatory, vasodilatory angiotensin 1–7 to reduce blood pressure (29). Overall, balanced ACE1/ACE2 regulation of angiotensin tone is central for blood pressure regulation. Advancing age may shift the ACE2-mediated homeostasis toward ACE1-driven inflammation and hypertension. Similarly, SARS-CoV-2 downregulates ACE2 activity through viral binding and internalization (30), shifting the balance toward dysregulated ACE1/angiotensin II signaling, vasoconstriction, and fibrosis (31, 32) (Figure 2).

#### 5.1.2 Age-related disparity in ACE1 and ACE2 expression

Age alters the ACE1 and ACE2 expression in tissues (33). Younger children have lower ACE2 expression in lung alveolar epithelium compared with older children and adults, which may explain lower rates of severe SARS-CoV-2 respiratory infections (34). However, from a vascular standpoint, ACE2 expression decreases with age. As mentioned earlier, the dominance of ACE1 signaling in older adults can lead to dysregulated inflammation and fibrosis in blood vessels, potentially contributing to higher hypertension risk (35). This provides a potential explanation for advanced age being a major risk factor for PCNH. As noted earlier, SARS-CoV-2 infection reduces endothelial ACE2 expression in blood vessels, which can exacerbate ACE1/ACE2 imbalance and shift the pathway toward further inflammation and fibrosis.

#### 5.1.3 Gender-related disparity in ACE1 and ACE2 expression

Females also have higher ACE2 expression compared with males. This is partly attributed to estrogen-enhancing ACE2 levels in various tissues, including lung alveolar cells. Additionally, the ACE2 gene is located on the X chromosome (36). While X chromosome inactivation generally silences most female genes, the process is incomplete for certain regions such as ACE2 (37). This overexpression helps explain the lower hypertension prevalence in females of fertile age. As noted by Gillis et al. (38), hypertension in the general population is more common in men. However, this gap narrows with age, likely due to declining ACE2 expression (39). Moreover, postmenopausal hypertension risk increases with dramatically reduced estrogen (39, 40).

Interestingly, despite higher protective ACE2 levels in females and more COVID-19 cases in men, post-COVID hypertension is more prevalent among women. Women also make up the majority of the COVID-19 long haulers (41). This paradox highlights the likely role of disproportionate immune activation and inflammation in driving this outcome in females (42). While women tend to have greater baseline ACE2 expression across age and race subgroups compared with males, SARS-CoV-2 infection may trigger a more drastic shift toward ACE1 dominance and away from vasoprotective ACE2 signaling. The resulting imbalance could initiate hypertension development. More severe ACE1/ACE2 distortion in infected women vs. men could explain the elevated risk of vascular complications such as hypertension. Additional research investigating sex differences in COVID-19 cardiovascular sequelae is still needed to unravel these complex mechanisms. Determining the contribution of both inflammation and angiotensin-converting enzyme (ACE) signaling dysregulation specifically in females will provide critical insights into the unexpectedly increased susceptibility of women to PCNH.

#### 5.1.4 Soluble ACE2 and autoantibodies against the soluble ACE2

ACE2 is a transmembrane protein that is originally located in the membrane of various cells. Studies have shown that several enzymes such as ADAM17 and TMPRSS2 can cleave the membrane-binding domain of ACE2, causing it to be released into the bloodstream. This form of ACE2 is known as soluble ACE2 (sACE2) (43).

Many observational studies have reported a positive association between the level of sACE2 in the blood and the severity of COVID-19 disease (44–46). Of note, sACE2 cannot inactivate angiotensin II (47). However, sACE2 can still serve as a receptor for SARS-CoV-2 (48). Interestingly, SARS-CoV-2 itself mediates ACE2 shedding and increases the sACE2 level in patients' serum (49). Therefore, we can assume that sACE2 facilitates SARS-CoV-2 spreading throughout the body and deteriorates the dysregulated inflammation caused by the virus.

On the other hand, studies have shown that some patients produce autoantibodies against soluble ACE2 (sACE2) after recovering from COVID-19. These autoantibodies can interfere with the function of both ACE2 and sACE2, which can lead to the overactivation of the angiotensin II/AT1 receptor pathway (50). This, in turn, could be a potential mechanism for PCNH. More research is needed to measure the levels of these autoantibodies in patients with and without PCNH to better understand the underlying mechanisms.

### 5.2 Inflammation

The role of inflammation in hypertension has been well-established in recent years (51–53). A recent study by Eberhardt et al. (54) on autopsies of eight patients who died as a result of severe COVID-19 infection demonstrated that SARS-CoV-2 has a tropism for vascular lesion macrophages and foam cells in the coronary vasculature, suggesting the role of the virus in triggering inflammation, particularly in the previously damaged areas. It is worth noting that macrophages and foam cells have a very low expression of ACE 2 and TMPRSS2. Therefore, SARS-CoV-2 infects these cells with a specific entry mechanism using a particular type of cell surface glucose-related protein (55).

Furthermore, in another study conducted by Chen et al. (22), patients with post-COVID new-onset hypertension exhibited significantly higher levels of troponin I (median interquartile 22, 95% CI: 18.20–30.00 vs. 3.86, 95% CI: 2.49–5.15) and procalcitonin (median interquartile 82, 95% CI: 53–430 vs. 49, 95% CI: 28–73), suggesting a potential inflammatory process. According to studies, the SARS-CoV-2 N-protein can activate NLRP3 inflammasomes, initiating an inflammatory cascade that plays a protective role against viral spread in the early stages. However, prolonged dysregulation of this pathway can damage the vasculature and lead to hypertension (56).

On the other hand, another major inflammatory pathway is primarily led by type I interferons (IFNs) which is inhibited by SARS-CoV-2. Studies have suggested that loss-of-function mutations in type I IFN or autoantibodies against type I IFN are associated with severe COVID-19 infections and increased mortality (57, 58). The type I interferon pathway appears to have less tendency toward prolonged hyperinflammation and subsequent hypertension (59). Therefore, we can infer that there is another balanced system between these two inflammatory pathways in the body, and dysregulation in either can shift toward hyperinflammation and hypertension.

Studies also demonstrated the crucial role of inflammation in the pulmonary vasculature in patients with COVID-19 infection (60). High levels of pro-inflammatory cytokines along with uncontrolled complement activation can lead to activation of coagulation pathways and microthrombi formation (61), all are correlated with the emergence of pulmonary hypertension in patients with COVID-19 infection (62, 63).

## 6 Genetic aspects of hypertension and COVID

Genome-wide association studies (GWAS) and Mendelian randomization (MR) methods elucidated the shared genetic structure between hypertension and COVID-19 infection (57, 64). Baranova et al. (57) analyzed the genome of hospitalized patients with severe COVID-19 infection and showed that patients with genetic liability to hypertension may have an increased risk of severe COVID-19 infection (OR = 1.05, *p* = 0.03. This may or may not be aligned to PCNH, given that many such patients may have been diagnosed with hypertension earlier. Nevertheless, the presence of a shared genetic structure between hypertension and severe COVID-19 enhances the likelihood that genetic factors may potentiate the development of PCNH in predisposed individuals. This study also highlighted a shared genetic structure linking the loci related to the immune system and blood group proteins to genetic susceptibility to COVID-19 infection. In other words, people harboring some variants in the genes related to the immune system and blood groups may experience more severe COVID-19 infection.

Additionally, another Mendelian randomization study found that genetic variants associated with COVID-19 at genome-wide significance were also associated with hypertensive disorders in pregnancy, using the inverse-variance weighted (IVW) and MR-Egger methods (OR = 1.11, *p* = 0.001) (58), suggesting a shared genetic architecture between hypertension and genetic liability to COVID-19 infection.

In support of this, Yang et al. (65) characterized the genetic landscape of ACE2—the receptor for SARS-CoV-2—and identified rare coding variants and regulatory polymorphisms that differ across sex and ancestry. Some of these variants may impair ACE2 expression or function, exacerbating RAS imbalance during infection. This functional depletion of ACE2 could tip the physiological axis toward vasoconstriction, inflammation, and hypertension, highlighting a plausible mechanism through which genetically susceptible individuals may develop post-COVID new-onset hypertension (PCNH).

Similarly, Faustine et al. (66) demonstrated that specific genotypes in the ACE (rs4331) and ACE2 (rs2074192) genes were associated with increased COVID-19 severity in patients with hypertension. Males with the GG (ACE) and TT (ACE2) genotypes experienced the highest rates of moderate-to-severe disease, suggesting that individuals with certain ACE/ACE2 polymorphisms may not only be at greater risk of severe infection but may also be more vulnerable to persistent RAS dysregulation and the development of PCNH.

Based on another research by Cheng et al., COVID-19 and hypertension genetic pathways can disproportionately affect females through the SPEG gene variant rs12474050. This genetic variant is associated with both severe COVID-19 outcomes and hypertension in women, suggesting that females may be at higher risk for developing PCNH. The study reveals that SPEG expression is naturally higher in female heart tissue and becomes further upregulated during SARS-CoV-2 infection, particularly in cardiomyocytes, which may explain why women experience more severe cardiovascular complications from COVID-19. This sex-specific genetic vulnerability could contribute to the observed increased incidence of new-onset hypertension in female COVID-19 patients compared with males (67).

## 7 Other potential causes of post-COVID new-onset hypertension

### 7.1 Obesity and metabolic syndrome

Obesity and related comorbidities are increasingly being viewed as a pandemic. During the COVID-19 outbreak, statistical analyses have indicated a substantial rise in the prevalence of obesity. Restrepo studied the average BMI of US citizens in 2020, compared with pre-pandemic data (68), the average BMI in US citizens increased by +0.6 (*p* < 0.05), and the obesity prevalence rates also increased by 3% (*p* < 0.05) (69). Aminian et al. (70) performed a study on 2,839 patients recovering from COVID-19 and found a significantly increased risk of cardiac (HR: 1.87, 95% confidence interval or CI: 1.41–2.48, *p* < 0.001) and vascular (HR: 2.43, 95% CI: 1.38–4.27, *p* = 0.13) complications among participants with a BMI over 35. This highlights the role of both preexisting and post-COVID-19 obesity in elevating the risk of PCNH.

### 7.2 Corticosteroids

This class of medications was widely used during the COVID-19 pandemic for both hospitalized and non-hospitalized patients (71). Although valid guidelines have approved their potential role in the outcome of critically ill patients (72, 73), studies show that these medications were overused in many countries and health systems (71). Hypertension accounts for one of the side effects of corticosteroid overuse among patients who recovered from a previous COVID-19 episode (64, 65). Corticosteroids can increase blood pressure by increasing the amount of salt and water that the body retains, although these effects may be transient. Corticosteroids can also reduce the production of nitric oxide, a molecule that contributes to vasodilation (10, 74, 75).

### 7.3 Physical inactivity

Physical inactivity is a well-known risk factor for hypertension (HR = 1.29, 95% CI: 1.01, 1.66) (76), and it has been more common among people during and after the COVID-19 pandemic (77). Analyses demonstrate that a decrease in physical activity during the pandemic involved all ages including the younger population (78). Physical inactivity can lead to weight gain, which is also another major risk factor for hypertension.

### 7.4 Closer monitoring postinfection

Greater engagement with the healthcare system among COVID-19 survivors may lead to the identification of previously undiagnosed conditions, such as hypertension (79).

## 8 Discussion

Post-COVID new-onset hypertension is a well-documented condition, reported in both hospital-based and population-based studies (77). In this review, we summarize the proposed mechanisms underlying PCNH, including the direct effects of the virus—primarily through ACE receptors and alterations in the renin–angiotensin system—as well as the roles of inflammation and endothelial injury. We also explore the contribution of genetic predisposition and the shared genetic architecture between severe COVID-19 and hypertension.

Recognizing the PCNH as a distinct clinical entity carries significant implications beyond academic interest. While the immediate management of elevated blood pressure might appear similar regardless of etiology, identifying PCNH enables targeted screening of COVID-19 survivors, particularly among traditionally low-risk populations such as younger females without conventional cardiovascular risk factors. Given the unprecedented scale of COVID-19 infection globally, even a modest increased risk of PCNH could translate into a substantial population-level burden of cardiovascular disease, particularly considering the younger age of onset compared with essential hypertension. This understanding should inform healthcare resource planning, as systems need to prepare for an increased burden of hypertension-related complications in previously low-risk populations.

Therefore, while we continue to investigate the exact mechanisms and optimal management strategies, the recognition and study of PCNH remain crucial for both individual patient care and broader public health preparedness strategies in the post-COVID era.

**Figure 3:**
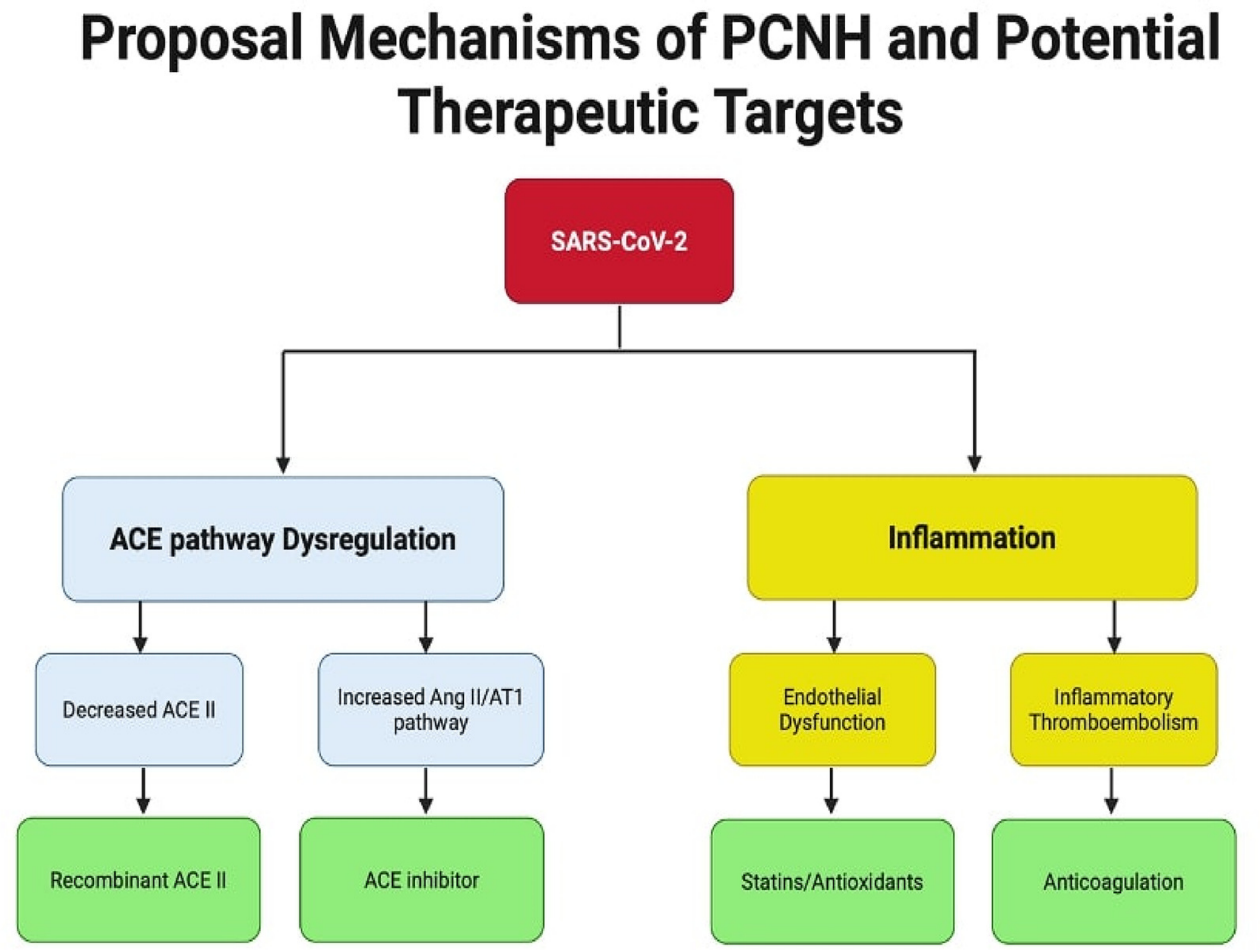
Summary of potential mechanisms of PCNH and therapeutic targets. Two main pathways contribute to post-COVID-19 new onset hypertension: ACE pathway dysregulation and inflammation. The ACE pathway leads to hypertension through either decreased ACE2 levels via receptor internalization or overactivation of the Ang II/AT1R pathway, ultimately causing fibrosis and inflammation. The inflammation pathway results directly from interleukin release and can also be triggered by AT1R pathway activation. Systemic inflammation promotes both endothelial dysfunction and hypercoagulable states, contributing to cardiovascular complications. ACE II, angiotension converting enzyme II; Ang II, angiotensin II; AT1R, angiotensin II type I receptor.

Despite the growing evidence supporting an association between COVID-19 and PCNH, several critical knowledge gaps warrant further investigation. The long-term natural history of PCNH remains poorly understood, while most current studies are limited to 6–12 months of follow-up. Moreover, while several mechanisms have been proposed, the relative contribution of each pathway particularly the ACE2 regulation, inflammation, and endothelial dysfunction to PCNH development remains unclear. The paradoxical higher prevalence in women despite their typically higher ACE2 expression requires more in-depth mechanistic investigation. Figure 3 presented potential therapeutic targets.

Additionally, standardized protocols for PCNH screening and early detection are lacking, as are predictive biomarkers for identifying high-risk patients. Future research priorities should include large-scale prospective cohort studies with extended follow-up periods, standardization of PCNH definition and diagnostic criteria, investigation of sex-specific mechanisms, and development of predictive models for early identification.

Furthermore, the establishment of large-scale, multicenter registries could help address many of these knowledge gaps while providing valuable data for clinical decision-making in the post-COVID era.

### 8.1 Study limitations and evidence quality

Current evidence is limited by heterogeneous PCNH definitions, variable follow-up periods, and potential confounding from lifestyle changes and increased healthcare utilization post-COVID. Most studies focus on hospitalized patients, potentially overestimating severity. Standardized diagnostic criteria and longer-term prospective studies with community-based populations are needed to better characterize this emerging condition.

## 9 Conclusion

In this review, we examined the prevalence, potential mechanisms, and public health significance of PCNH. Studies have linked COVID-19 infection with the development of hypertension as well as the worsening of preexisting hypertension. Proposed causal mechanisms for this link include dysregulated ACE1/ACE2 balance, inflammation, soluble ACE2 autoantibodies, and shared genetic susceptibility between the conditions. Other potential causes of PCNH, such as stress, anxiety, obesity, metabolic syndrome, physical inactivity, and corticosteroid side effects, have also been implicated. The long-term progression, natural course, and optimal management of PCNH, beyond conventional therapies, are not yet well understood and warrant further investigations.
